# Supplementary material for: PEDLA: predicting enhancers with a deep learning-based algorithmic framework
Source: Sci Rep. 2016 Jun 22;6:28517. doi: 10.1038/srep28517 (PMC4916453; doi:10.1038/srep28517)
Supplement: Supplementary Information [file srep28517-s1.pdf]

Supplementary materials for

PEDLA: predicting enhancers with deep learning-based algorithmic framework

Feng Liu[1], Hao Li[1], Chao Ren[1], Xiaochen Bo[1*], Wenjie Shu[1*]

[1]Department of Biotechnology, Beijing Institute of Radiation Medicine, Beijing 100850, China

[*]Corresponding author. To whom correspondence should be addressed. Tel & Fax: +86 10 68210077 66932211; Email: shuwj@bmi.ac.cn. Correspondence may also be addressed to: boxc@bmi.ac.cn.

Supplementary Methods

Deep belief networks and hidden Markov model

Deep belief networks (DBNs), initially introduced by Hinton, et al., [1], are probabilistic generative models that are in contrast to the discriminative nature of traditional neutral nets. DBNs consist of several layers of Restricted Boltzmann Machines (RBMs) [2], which are a type of undirected bipartite graph constructed from a bottom layer of binary stochastic hidden units $\mathbf{h}$ and a top layer of stochastic visible units $\mathbf{v}$. For an RBM, an energy function is assigned to the configurations of $\mathbf{v}$ and $\mathbf{h}$ in terms of

$$\mathrm{E}(\mathbf{v},\mathbf{h}) = -\mathbf{b}^{\mathrm{T}}\mathbf{v} - \mathbf{c}^{\mathrm{T}}\mathbf{h} - \mathbf{v}^{\mathrm{T}}\mathbf{W}\mathbf{h}$$

where $\mathbf{W}$ is the symmetrical matrix of visible/hidden connection weights and $\mathbf{b}$ and $\mathbf{c}$ are the biases of the visible and hidden units, respectively. Thus, the probability distribution of any particular setting of $\mathbf{v}$ and $\mathbf{h}$ is

$$\mathrm{P}(\mathbf{v},\mathbf{h}) = \frac{e^{-E(\mathbf{v},\mathbf{h})}}{Z}$$

where the normalization factor $\mathrm{Z} = \sum_{\mathbf{v},\mathbf{h}} e^{-E(\mathbf{v},\mathbf{h})}$ is known as the partition function. The bipartite and binary natures of RBMs enable us to derive simple exact expression for $\mathrm{P}(\mathbf{v}|\mathbf{h})$ and $\mathrm{P}(\mathbf{h}|\mathbf{v})$ as

$$\mathrm{P}(\mathbf{h} = \mathbf{1}|\mathbf{v}) = \sigma\,(\mathbf{c} + \mathbf{v}^{\mathrm{T}}\mathbf{W})$$

and

$$\mathrm{P}(\mathbf{v} = \mathbf{1}|\mathbf{h}) = \sigma\,(\mathbf{b} + \mathbf{h}^{\mathrm{T}}\mathbf{W}^{\mathrm{T}})$$

, respectively, where σ denotes the (elementwise) logistic sigmoid, $\sigma\,(x) = (1 + e^{-x})^{-1}$.

In our algorithm, RBMs were trained in a greedy layer-wise manner with one-step contrastive divergence (CD-1). We used the DBN weights resulting from RBMs to initialize DNNs generatively in a purely unsupervised way and used the outputs of DBN as the inputs to train the Softmax output layer in a supervised manner. After pre-training, we used a backpropagation algorithm to fine-tune all of the weights in a supervised manner to improve the discriminative performance of the entire network. Pre-training followed by stochastic gradient descent is used to train DNN because it often outperforms random initialization for the deeper architectures and provides robust results to the initial random seed. Studies have illustrated that using DBN pre-training to initialize the weights of a DNN helps prevent overfitting and can aid in subsequent optimization and can reduce generalization error [3, 4]. This semi-supervised approach using deep models has proved effective in a number of applications, including coding and classification for speech, audio, text, and image data.

An hidden Markov model (HMM) is a generative model in which the system is assumed to be generated from a Markov process that transitions between states $\boldsymbol{S} = [s_1, \ldots, s_K]$. An HMM is a triple $(\boldsymbol{\pi}, \mathbf{A}, \mathbf{B})$, where $\boldsymbol{\pi}$ is the initial state probability distribution, $\mathbf{A}$ is the state transition probability distribution and $\mathbf{B}$ is the observation probability distribution. For an HMM, $\boldsymbol{B}$ is defined as

$$b_j(O_t) = P\left(O_t \middle| q_t = S_j\right) = \frac{p\left(q_t = S_j \middle| O_t\right) p(O_t)}{p(q_t = S_j)},\ 1 \le j \le N,$$

where O_t is the observation at location *t*, q_t is the state at location *t*, and S_j is the *j*-th state of the *N* states in total, $p\left(q_t = S_j \middle| O_t\right)$ is the state posterior probability, and

$p(q_t = S_j)$ is the prior probability of each state.

In our hybrid model, $p\left(q_t = S_j \middle| O_t\right)$ is estimated from the DNN, $p(q_t = S_j)$ can be easily estimated from the training set, and $p(O_t)$ is independent of state and thus can be ignored without any influence on the result when using the Viterbi algorithm to find the optimal state. Notably, we have found the prior probability $p(q_t = S_j)$ to be very important in alleviating the label bias problem.

References

1. Hinton, G.E., Osindero, S. & Teh, Y.W. A fast learning algorithm for deep belief nets. *Neural computation* **18**, 1527-1554 (2006).
2. Hinton, G. Deep belief networks. *Scholarpedia* **4**, 5947 (2009).
3. Hinton, G.E. & Salakhutdinov, R.R. Reducing the dimensionality of data with neural networks. *Science* **313**, 504-507 (2006).
4. Erhan, D. et al. Why Does Unsupervised Pre-training Help Deep Learning? *J. Mach. Learn. Res.* **11**, 625-660 (2010).

Supplementary Figures

Supplementary Figure S1. The workflow of PEDLA

Schematic diagram showing the input data (A), training procedure (B) and prediction procedure (C) of PEDLA.

Supplementary Figure S2. Performance of classic DNN to handle class-imbalanced data

(A-B) Inability to handle class-imbalanced data in an unbiased way for classic DNN. Three performance indicators, sensitivity, specificity and GM, were measured for the training set (A) and test set (B) using 5-fold cross-validation based on the optimal structure of classic DNN with all 1,114-dimensional features. The numbers of enhancers, promoters and random regions not annotated as promoters or enhancers were maintained at $1 : 1 : x$ ($x = 1, 2, \ldots, 9$), such that the ratio between positive and negative samples was $1 : (1 + x)$.

Supplementary Tables

Supplementary Table S1. Description of all 1,114-dimensional feature data used by PEDLA in the H1 cell line.

Supplementary Table S2. The enhancer set (positive class set) containing 5,870 enhancer regions based on H3K27ac peaks in the H1 cell line.

Supplementary Table S3. Performance comparisons of various structures of PEDLA using 5-fold cross-validation in both the training set and test set.

Supplementary Table S4. Twenty-two training cell types/tissues and twenty independent test cell types/tissues selected for training and evaluation of PEDLA.

Supplementary Table S5. Variable importance of all 1,114-dimensional features.

We used the random forest method to assess the relative importance of each feature by measuring the increase in classification error upon permutation of feature values across classes.

Supplementary Table S6. Performance comparisons across methods with less stringent positive enhancer sets *p*-value $< 10^{-4}$

Supplementary Table S7. Performance assessments of PEDLA with the best-trained model in the 22 training cell types/tissues.

Supplementary Table S8. Performance assessments of PEDLA with the best-trained model in the 20 independent test cell types/tissues.

Figure S1

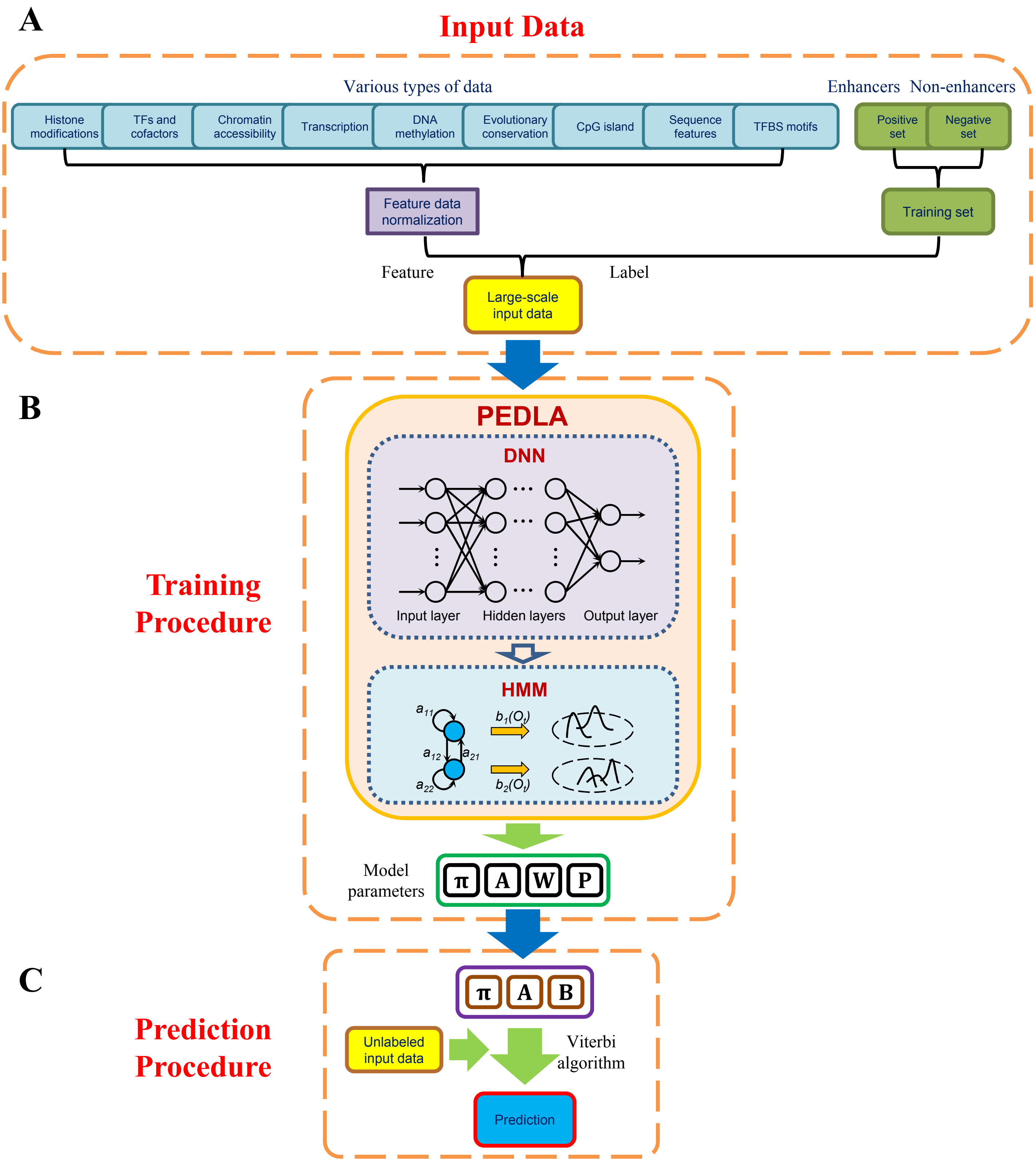
A
Input Data
Various types of data
Enhancers Non-enhancers
Histone modifications
TFs and cofactors
Chromatin accessibility
Transcription
DNA methylation
Evolutionary conservation
CpG island
Sequence features
TFBS motifs
Positive set
Negative set
Feature data normalization
Training set
Feature
Label
Large-scale input data
B
Training Procedure
PEDLA
DNN
Input layer
Hidden layers
Output layer
HMM
a_{11}
a_{12}
a_{21}
a_{22}
$b_1(O_t)$
$b_2(O_t)$
Model parameters
π A W P
C
Prediction Procedure
π A B
Unlabeled input data
Viterbi algorithm
Prediction

Figure S2

A

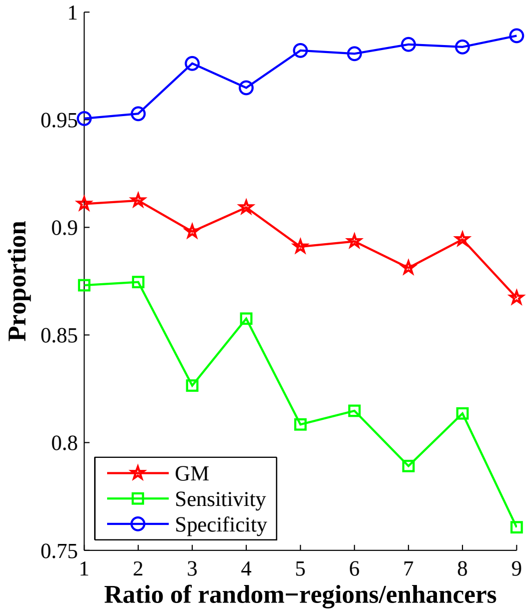

1
0.95
0.9
0.85
0.8
0.75
Proportion
1
2
3
4
5
6
7
8
9
Ratio of random−regions/enhancers
GM
Sensitivity
Specificity

B

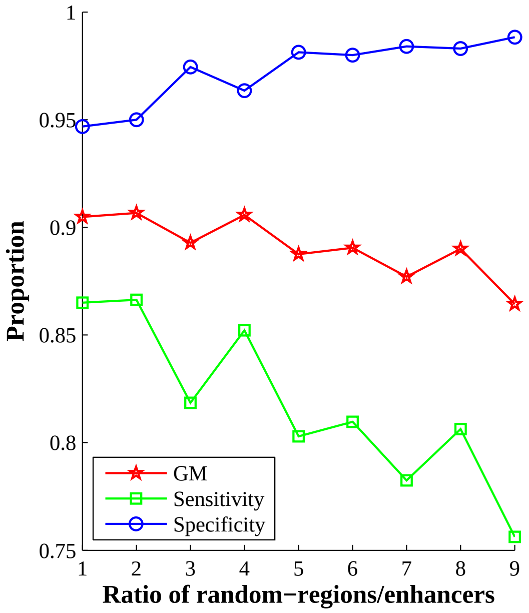

1
0.95
0.9
0.85
0.8
0.75
Proportion
1
2
3
4
5
6
7
8
9
Ratio of random−regions/enhancers
GM
Sensitivity
Specificity
